# Hippocampal-prefrontal theta-gamma coupling during performance of a spatial working memory task

**DOI:** 10.1038/s41467-017-02108-9

**Published:** 2017-12-19

**Authors:** Makoto Tamura, Timothy J. Spellman, Andrew M. Rosen, Joseph A. Gogos, Joshua A. Gordon

**Affiliations:** 1 Department of Psychiatry, Columbia University, New York, NY 10032 USA; 2 Neuroscience Research Unit, Mitsubishi Tanabe Pharma Corporation, Yokohama, Kanagawa 227-0033 Japan; 3 Department of Physiology and Cellular Biophysics, Columbia University, New York, NY 10032 USA; 4 Department of Neuroscience, Columbia University, New York, NY 10032 USA; 5 Division of Integrative Neuroscience, New York State Psychiatry Institute, New York, NY 10032 USA; 6 Present Address: National Institute of Mental Health, Bethesda, MD 20892 USA

## Abstract

Cross-frequency coupling supports the organization of brain rhythms and is present during a range of cognitive functions. However, little is known about whether and how long-range cross-frequency coupling across distant brain regions subserves working memory. Here we report that theta–slow gamma coupling between the hippocampus and medial prefrontal cortex (mPFC) is augmented in a genetic mouse model of cognitive dysfunction. This increased cross-frequency coupling is observed specifically when the mice successfully perform a spatial working memory task. In wild-type mice, increasing task difficulty by introducing a long delay or by optogenetically interfering with encoding, also increases theta–gamma coupling during correct trials. Finally, epochs of high hippocampal theta–prefrontal slow gamma coupling are associated with increased synchronization of neurons within the mPFC. These findings suggest that enhancement of theta–slow gamma coupling reflects a compensatory mechanism to maintain spatial working memory performance in the setting of increased difficulty.

## Abstract (web summary)

Theta- and gamma-frequency oscillatory synchrony correlates with spatial working memory performance. Here the authors report increases in theta-gamma cross-frequency coupling as a compensatory mechism associated with better working memory performance in models of cognitive dysfunction in mice.

## Introduction

Long-range functional connectivity is thought to contribute to a variety of higher brain functions, including learning and memory^1^, attention^2^, emotion^3^, motor behavior^4^, and working memory^5^. Considerable evidence links this long-range connectivity to oscillations in various frequency ranges^6, 7^. Oscillations represent cyclical fluctuations in neural activity within a brain region; functional connectivity is inferred when these fluctuations appear synchronized in two or more brain regions over time. There is accumulating evidence for a relationship between synchronous oscillatory activity and behavior^1, 8, 9^. Increasingly, it has become clear that there is also interaction between oscillations at different frequencies; for example, one can show that gamma-frequency (30–120 Hz) oscillations are stronger at particular phases of the theta-frequency (4–12 Hz) cycle, either within a brain region^10–14^ or across brain regions^15, 16^, and in a variety of behavioral tasks and species^17–20^.

The link between long-range synchrony and behavior has been studied extensively in rodent models. Many single neurons in the medial prefrontal cortex (mPFC) are modulated by theta-frequency oscillations in the hippocampus^8, 21, 22^. This theta-frequency synchrony between the hippocampus and PFC is associated with successful spatial working memory performance^23–26^. Additionally, recent evidence suggests that gamma-frequency synchrony between the hippocampus and other connected brain regions also contributes to spatial working memory^27, 28^. Yet despite the considerable evidence for cross-frequency coupling between theta and gamma oscillations^29^, little attention has been paid to the role of such coupling in spatial working memory.

In this study, the role of cross-frequency coupling in spatial working memory is examined using several approaches. The findings presented here demonstrate that slow gamma (30–70 Hz) oscillations in the mPFC are coupled to theta oscillations both locally and in the ventral hippocampus (vHPC). This theta–gamma coupling is enhanced in a genetic mouse model of cognitive dysfunction. Furthermore, this enhancement is associated with successful task performance in the setting of increased difficulty, an association that is confirmed using both behavioral and optogenetic manipulations. Finally, increases in the strength of coupling appear to drive increases in firing rate and synchrony of mPFC neurons. Together these results suggest that increases in vHPC theta-mPFC slow gamma synchrony underlie successful behavioral performance in the setting of increases in task difficulty, reflecting a potential circuit-based compensatory mechanism important for effective spatial working memory behavior.

## Results

### Cross-frequency coupling between vHPC theta and mPFC gamma

**Fig. 1:**
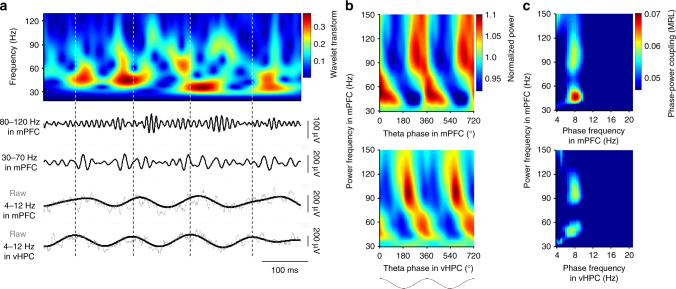
Hippocampal theta oscillations modulate prefrontal gamma oscillations. **a** Top: an example wavelet transform of local field potentials in the mPFC. Bottom: fast (80–120 Hz) and slow (30–70 Hz) gamma oscillations in the mPFC and raw LFP and theta (4–12 Hz) oscillations in the mPFC and vHPC. Dashed lines illustrate peaks of vHPC theta cycles. **b** Color-coded gamma power in the mPFC as a function of theta phase in the mPFC (top) and vHPC (bottom). Fast and slow gamma oscillations in the mPFC were seen coupled with distinct phases of theta oscillations in both of the vHPC and mPFC. **c** Phase-power comodulogram between fast-frequency power in the mPFC and low-frequency oscillation phase in the mPFC (top) and vHPC (bottom), demonstrating both of prefrontal and ventral hippocampal theta oscillations modulate the two separate ranges of gamma power in the mPFC

To investigate the dynamics of long-range cross-frequency coupling during performance of a spatial working memory task, mice were chronically implanted with microelectrode in the mPFC and dorsal and ventral hippocampus (dHPC and vHPC; Supplementary Fig. 1). As has been shown previously^30^, local field potentials (LFPs) recorded from within the mPFC have prominent theta and gamma oscillations that can be revealed by wavelet transforms or bandpass filters (Fig. 1a, top and bottom, respectively). The gamma component can be dissociated into separate bursts of slow (30–70 Hz) and fast (80–120 Hz) gamma, which occur most frequently at the peak and ascending phase of the local theta oscillation, respectively (Fig. 1a, b, top). In the mPFC, slow and fast gamma are strongly coupled to mPFC theta-frequency oscillations; both peaks clearly stand out in comodulograms that visualize the strength of coupling (warmer colors = stronger coupling) as a function of the two frequencies (Fig. 1c, top).

As others have shown^15^, cross-frequency coupling can occur across distant sites, in addition to locally as described above. One of the major inputs to the mPFC is the vHPC, and both slow and fast gamma oscillations in the mPFC are concentrated on specific phases of the vHPC theta cycle (Fig. 1a, b, bottom). The phase and frequency characteristics of this long-distance cross-frequency coupling are remarkably similar to those of coupling within the mPFC (Fig. 1b, c), though both mPFC gamma components are more strongly coupled to local theta than to vHPC theta (compare top and bottom plots in Fig. 1b, c).

### Altered coupling in a mouse model of cognitive dysfunction

**Fig. 2:**
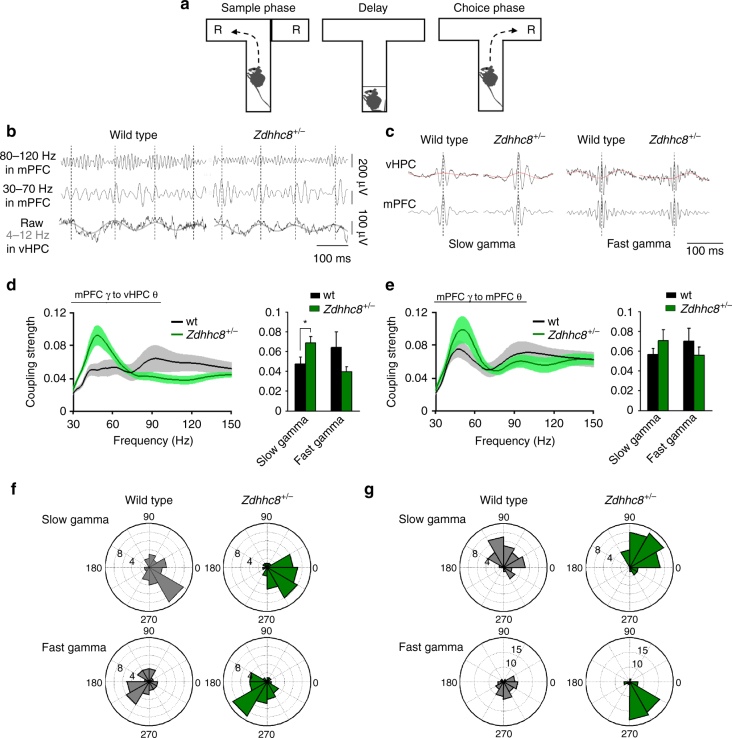
Theta–slow gamma coupling between vHPC and mPFC is augmented in *Zdhhc8* ^*+/−*^ mice. **a** Delayed non match to sample T-maze task. Each trial of the task comprised a sample phase and choice phase separated by 10 s delay. R: reward. The mouse images were created by the authors. **b** Example traces of fast gamma- and slow gamma-range filtered local field potentials in the mPFC and theta-ranged filtered local field potentials in the vHPC. Left: wild type. Right: *Zdhhc8* ^*+/−*^. **c** Averaged raw (black) and theta-range filtered (red) traces in the vHPC (top) obtained by aligning the signals at the peak of slow (left) and fast (right) gamma oscillations in the mPFC (bottom). **d** Left: strength of vHPC theta modulation of mPFC gamma as a function of power frequency in the mPFC. Right: averaged theta-gamma coupling in the slow gamma–range (theta–slow gamma coupling) and fast gamma range (theta–fast gamma coupling), demonstrating significantly higher theta–slow gamma coupling in *Zdhhc8* ^*+/−*^ mice. On the other hand, this mutant showed weaker vHPC theta-mPFC fast gamma coupling, though it did not reach statistical significance (slow gamma; *p* = 0.044, fast gamma; *p* = 0.14). *p < 0.05. Student’s *t*-test. *n* = 8 wild-type (wt) and *n* = 9 *Zdhhc8* ^*+/−*^ mice. **e** Left: strength of coupling of mPFC gamma with local mPFC theta as a function of power frequency in the mPFC. Right: averaged theta–gamma coupling in the slow and fast gamma range. Theta–gamma coupling did not differ by genotype (slow gamma; *p* = 0.29, fast gamma; *p* = 0.35). Student’s *t*-test. **f**, **g** Distribution of the mean direction of gamma power in the mPFC for the theta cycle phase in the vHPC (**f**) and mPFC (**g**) for 32 recordings from 8 wild type and 33 recordings from 9 *Zdhhc8* ^*+/−*^ mice. Mean direction did not significantly differ by genotype regardless of gamma range (slow gamma to vHPC theta; *p* = 0.98, fast gamma to vHPC theta; *p* = 0.12, slow gamma to mPFC theta; *p* = 0.11, fast gamma to mPFC theta; *p* = 0.85). Watson–Williams test

To examine the relationship between cross-frequency coupling and cognitive function, coupling was examined in the center arm of T-maze during performance of a delayed-non-match-to-place (DNMTP) task^25^ (Fig. 2a). This task involves a sample phase, in which encoding of goal location occurs, and a choice phase, in which retrieval and action selection occurs, guided by the remembered sample goal location. We reasoned that if theta–gamma coupling were functionally related to T-maze task performance, coupling might be disturbed in mice impaired at this task.

We have previously shown that mice carrying a heterozygous deletion of *Zdhhc8* (*Zdhhc8* ^+/−^ mice), a gene within the 22q11.2 microdeletion region that contributes to cognitive dysfunction and schizophrenia^31^, demonstrate a range of phenotypes, including impaired acquisition of the T-maze DNMTP task, and reduced theta-frequency synchrony between the vHPC and mPFC^32^ (Supplementary Fig. 2). Recordings obtained from *Zdhhc8* ^+/−^ mice (*n* = 9) and wild-type littermates (*n* = 8) showed that long-range cross-frequency coupling between the vHPC and mPFC was indeed disturbed in *Zdhhc8* ^+/−^ mice, though in a surprising manner (Supplementary Fig. 3). Both wild-type and mutant mice had prominent fast and slow gamma-frequency oscillations in the mPFC (Fig. 2b, c, Supplementary Fig. 3b). The strength of coupling of these oscillations to vHPC theta varied by genotype and frequency (Fig. 2d); slow gamma power varied more strongly as a function of theta phase in *Zdhhc8* ^+/−^ mice compared to wild types. By contrast, fast gamma power modulation with theta phase was weaker in the mutants, though this decrease did not reach statistical significance. This effect was not apparent in coupling of mPFC gamma with local mPFC theta; no significant differences were seen in either gamma range (Fig. 2e). These phenomena could not be accounted for by electrode positions (Supplementary Fig. 4) or running speed (data not shown). While the strength of vHPC theta–mPFC gamma modulation was altered, the temporal dynamics was left intact. The theta phase at which gamma strength was highest did not differ by genotype (Fig. 2f, g). Also, there were no significant differences in the shape, asymmetry or phase shifting of theta frequency oscillations by genotype (Supplementary Figs. 5 and 6). Phase–phase coupling, which reflects the timing but not the strength of the relationship between the two oscillations^14, 16^, was also unchanged (Supplementary Figs. 7 and 8). Computation of theta–gamma coupling in other pairs of recorded regions (mPFC–dHPC, vHPC–dHPC, vHPC–vHPC and dHPC–dHPC) revealed that there were not any significant differences in coupling strength by genotype, regardless of frequency range (Supplementary Fig. 9 for mPFC–dHPC coupling, data not shown for others). These data demonstrate augmented strength of vHPC theta–mPFC slow gamma coupling in *Zdhhc8* ^*+/−*^ mice without any alteration in the temporal alignment of the two oscillations.

**Fig. 3:**
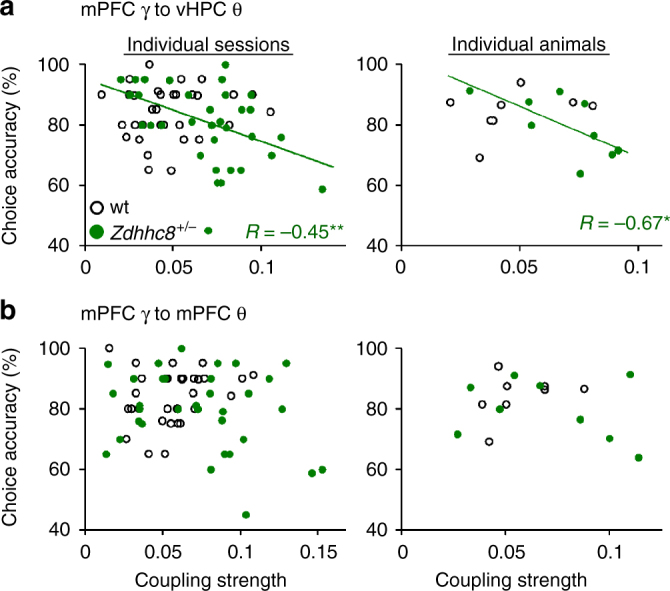
vHPC theta–mPFC slow gamma coupling in *Zdhhc8* ^*+/−*^ mice is inversely correlated with working memory performance. **a** Scatter plots depicting choice accuracy in a working memory task plotted against coupling strength between mPFC slow gamma and vHPC theta. Left: each session, right: each animal. Only *Zdhhc8* ^*+/−*^ mice showed significant inverse correlation of choice accuracy with coupling strength (Individual sessions: wt; *R* = 0.14, *p* = 0.43, *Zdhhc8* ^*+/−*^; *R* = −0.45, *p* = 0.0089. Individual animals: wt; *R* = 0.34, *p* = 0.41, *Zdhhc8* ^*+/−*^; *R* = −0.67, *p* = 0.049). *R*: correlation coefficient. *p < 0.05. **p < 0.01; Pearson’s correlation. **b** Choice accuracy did not correlate with theta-slow gamma coupling within mPFC. (Individual sessions: wt; *R* = 0.21, *p* = 0.20, *Zdhhc8* ^*+/−*^; *R* = −0.25, *p* = 0.15. Individual animals: wt; *R* = 0.36, *p* = 0.38, *Zdhhc8* ^*+/−*^; *R* = −0.27, *p* = 0.48). *R*: correlation coefficient, Pearson’s correlation

**Fig. 4:**
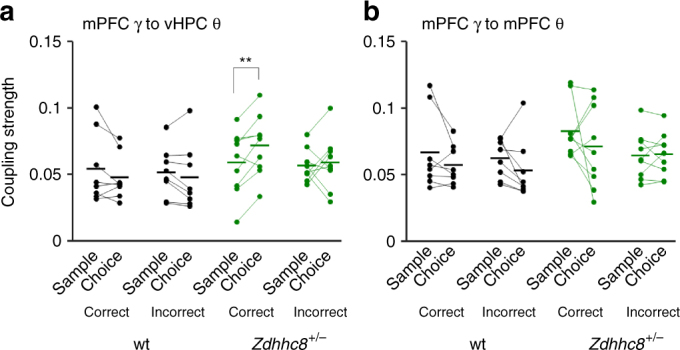
vHPC theta–mPFC slow gamma coupling in *Zdhhc8* ^*+/−*^ mice is enhanced with successful task execution of spatial working memory. **a** Strength of theta–slow gamma coupling in sample and choice phases in correct and incorrect trials. *Zdhhc8* ^*+/−*^ mice showed significantly stronger coupling in choice than sample phases of correct trials but not incorrect trials, whereas wild-type mice showed equivalent coupling between sample and choice phases. (correct trials, wt; *p* = 0.17, *Zdhhc8* ^*+/−*^; *p* = 0.0078, incorrect trials, wt; *p* = 0.26, *Zdhhc8* ^*+/−*^; *p* = 0.71). **p < 0.01; Paired *t*-test. **b** Coupling strength of mPFC gamma with local mPFC theta did not change in a task dependent manner. Correct trials, wt; *p* = 0.22, *Zdhhc8* ^*+/−*^; *p* = 0.35. Incorrect trials, wt; *p* = 0.21, *Zdhhc8* ^*+/−*^; *p* = 0.28

Careful examination of the relationship between cross-frequency coupling and behavior suggests that the enhancement in theta–gamma coupling is associated with successful task execution, despite being elevated in a model of deficient task acquisition. Consistent with our previous results demonstrated that genotype-associated changes in vHPC–mPFC synchrony are greatest in animals with the greatest deficits in behavior^27^, decreased choice accuracy was associated with higher levels of vHPC theta–mPFC slow gamma coupling in the center arm of the maze in the choice phase, in the *Zdhhc8* ^*+/−*^ group, but not in wild types (Fig. 3a). This was true both on a session-by-session and animal-by-animal basis (Fig. 3a). Within-trial and trial-by-trial analyses, however, revealed something quite different. In the very same animals, vHPC theta–mPFC slow gamma coupling was greater in the choice phase than the sample phase of the task; this phase-specific increase was present only in correct trials; no such difference was found during incorrect trials (Fig. 4a). These seemingly paradoxical results suggest that for the mutants, successfully executing the task involves a compensatory increase in theta–gamma coupling between the vHPC and mPFC, specifically during the choice phase. This compensatory increase comes against a background of reduced coupling.

Task-induced enhancement of theta–slow gamma coupling was highly specific, as local cross-frequency coupling between mPFC theta and mPFC slow gamma did not differ by task phase (Fig. 4b), nor did it correlate with behavioral performance (Fig. 3b). vHPC theta–mPFC fast gamma coupling also did not show any association with behavioral performance (Supplementary Fig. 10).

Altered long-range cross-frequency coupling was not explained by differences in power or coherence. Oscillatory power did not significantly differ by genotype, regardless of region or frequency, nor did slow or fast gamma power correlate with cross-frequency coupling (Supplementary Fig. 3). While *Zdhhc8* ^*+/−*^ mice have decreased theta coherence between the vHPC and mPFC^32^ (Supplementary Fig. 2c), theta coherence did not correlate with theta–slow gamma coupling (Supplementary Fig. 11a). Finally, because coherence in the slow gamma range was slightly (but not significantly) elevated in *Zdhhc8* ^*+/−*^ mice (Supplementary Fig. 2c), the relationship between slow gamma coherence, theta–gamma coupling, and behavior was specifically probed. Slow gamma coherence between the vHPC and mPFC correlated with vHPC theta–mPFC slow gamma coupling in *Zhhc8* ^*+/−*^ mice, but did not differ between correct and incorrect trials (Supplementary Fig. 11b, c). Taken together, these demonstrate a robust correlation between vHPC theta–mPFC slow gamma coupling, a mouse model of cognitive dysfunction, and spatial working memory performance.

### Theta–gamma coupling is modulated by task difficulty

The association between enhanced long-range cross-frequency coupling and correct trials suggests that increased coupling might be associated with successful task completion in the setting of increased difficulty. We tested this prediction in two different ways.

**Fig. 5:**
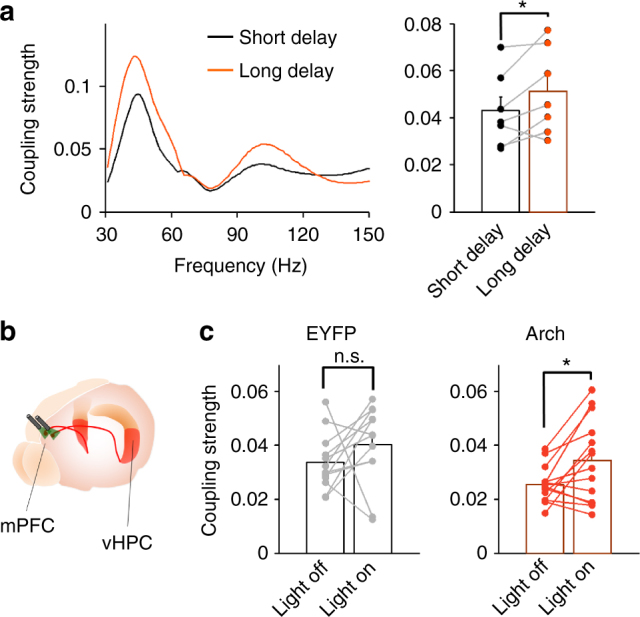
vHPC theta–mPFC slow gamma coupling is enhanced with the challenge of a working memory task. **a** Left: example plot demonstrating theta–gamma coupling in short-delay (black) and long-delay (orange) trials. Right: averaged coupling strength in short- and long-delay trials. Coupling between mPFC slow gamma and vHPC theta was significantly higher in long-delay trials than short delay trials. *p < 0.05; paired *t*-test. **b** Schematic of optogenetic inhibition of ventral hippocampal–prefrontal connectivity. Arch-encoding viruses or EYFP-encoding control viruses were injected into the vHPC. vHPC axonal terminals in the mPFC was specifically illuminated to silence mPFC-projecting vHPC neurons. **c** vHPC theta–mPFC slow gamma coupling in correct trials of control viruses or Arch viruses injected mice. Silencing of vHPC–mPFC projection significantly enhanced theta–slow gamma coupling (EYFP; *p* = 0.24, Arch; *p* = 0.019). *p < 0.05; paired *t*-test. n.s.: not significant

First, we increased task difficulty by inducing a longer delay between the sample and choice phases of the task (Supplementary Fig. 12). Accordingly, vHPC theta–mPFC slow gamma coupling was compared between short delay (10 s) and long delay (90 s) trials in wild-type mice. Coupling was significantly higher in long-delay than short-delay trials (Fig. 5a).

Second, we have previously demonstrated that performance on the T-maze task is impaired by optogenetic inhibition of vHPC-to-mPFC terminals^27^ (Fig. 5b, Supplementary Fig. 12). Specifically, inhibition of these terminals during the sample phase impairs performance on the subsequent choice phase. Here data from the choice phase of correct trials was re-analyzed for theta–gamma coupling strength, comparing trials in which the terminals had been inhibited during the sample to those in which there had been no inhibition. vHPC theta–mPFC slow gamma coupling was stronger after terminal inhibition, only in animals expressing the inhibitory opsin, and not in animals expressing a control flourophore (Fig. 5c, light; F_1,48_ = 4.2, *p* = 0.047, opsin; F_1,48_ = 2.5, *p* = 0.12, light × opsin; F_1,48_ = 1.09, *p* = 0.30, two-way ANOVA). Running speed was not affected by terminal inhibition. This effect was not observed when the terminals were directly inhibited during the choice (Arch mice: Light off; 0.025 ± 0.0026, Light on; 0.033 ± 0.0040, *p* = 0.12), and thus did not represent a direct effect of terminal inhibition.

Finally, we examined theta-gamma coupling in an additional mutant mouse line. Augmented theta–gamma coupling was also obtained from another mouse model of cognitive dysfunction, *Dgcr8* ^*+/−*^ mice^33^. Similar to *Zhhc8* ^*+/−*^ mice, vHPC theta–mPFC slow gamma coupling in correct trials was significantly higher in mutant mice than wild-type mice without affecting vHPC theta–mPFC fast gamma coupling (Supplementary Fig. 13).

Taken together, these data lend further support to the notion that long-range theta–gamma coupling between the vHPC and mPFC reflects a compensatory mechanism to maintain behavioral performance in the setting of increased difficulty.

### Theta–gamma coupling synchronizes mPFC neuronal activity

**Fig. 6:**
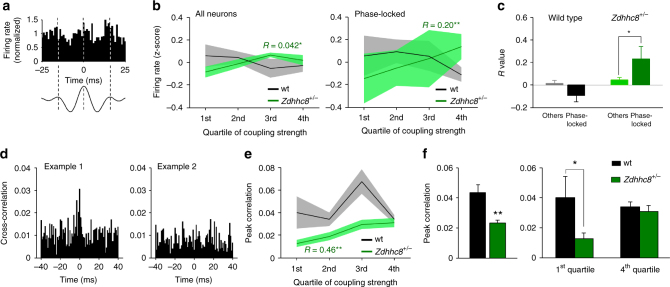
vHPC theta–mPFC slow gamma coupling drives mPFC synchrony in *Zdhhc8* ^*+/−*^ mice. **a** Example firing rate of an mPFC single unit obtained by aligning at the peak of slow gamma oscillations in the mPFC. Dashed lines illustrate peaks of gamma cycles. **b** Firing rates as a function of strength of vHPC theta–mPFC slow gamma coupling for all neurons (left) and significantly phase-locked neurons (right). Z-score of firing rate was averaged within animals, then across animals. Coupling strength was positively correlated with firing rate of both of phase-locked and all neurons in *Zdhhc8* ^*+/−*^ mice (all neurons: wt; *p* = 0.52, *Zdhhc8* ^*+/−*^; *p* = 0.029, Phase-locked neurons: wt; *p* = 0.157, *Zdhhc8* ^*+/−*^; *p* = 0.00094). *R*: correlation coefficient. **p < 0.01, *p < 0.05; multiple regression analysis. *n* = 288 neurons from 8 wild type and *n* = 293 from 8 *Zdhhc8* ^*+/−*^ mice. **c** Mean correlation coefficient of firing rates and vHPC theta–mPFC slow gamma coupling for significantly phase-locked neurons and non-significantly phase-locked neurons (Others). Positive correlation between firing rate and vHPC theta–mPFC slow gamma coupling was significantly higher in phase-locked neurons of *Zdhhc8* ^*+/−*^ mice. This tendency was not the case in wild-type control mice (wt; *p* = 0.15, *Zdhhc8* ^*+/−*^; *p* = 0.047). *p < 0.05; Student’s *t*-test. **d** Example spike train cross-correlograms. Example 1 shows relatively higher synchrony of firing than example 2. **e** Spike cross-correlogram as a function of coupling strength. vHPC theta–mPFC slow gamma coupling was positively correlated with spike cross-correlogram only in *Zdhhc8* ^*+/−*^ mice. **p < 0.01; multiple regression analysis (wt; *p* = 0.52, *Zdhhc8* ^*+/−*^; *p* = 0.0022). *n* = 30 pairs from 5 wild type and 49 pairs from 6 *Zdhhc8* ^*+/−*^ mice. **f** Mean spike cross-correlogram. Left: overall spike cross-correlogram was significantly lower in *Zdhhc8* ^*+/−*^ mice. This was reversed in higher theta–slow gamma state (Right). *p < 0.05,**p < 0.01; Student’s *t*-test

In order to affect behavior, vHPC theta–mPFC slow gamma coupling must presumably influence the activity of neurons in the mPFC. The relationship of vHPC theta–mPFC slow gamma coupling with activity and spike timing of mPFC single units was therefore examined in both wild-type and *Zdhhc8* ^*+/−*^ mice. Overall firing rates were significantly higher in *Zdhhc8* ^*+/−*^ mice than wild types (Supplementary Fig. 14c). In a subset of neurons from mice of either genotype, firing rate was higher on particular phases of the mPFC slow gamma oscillation; neurons are considered “phase-locked” if this tendency is greater than expected by chance (Fig. 6a; Supplementary Fig. 14a, b). Overall, fewer units were phase-locked to mPFC slow gamma in *Zdhhc8* ^*+/−*^ mice that in wild types, though the difference did not reach statistical significance (Supplementary Fig. 14a, b). To examine the relationship between firing rate and vHPC theta–mPFC gamma coupling, sessions were divided into epochs where the strength of this coupling ranged from low to high (in quartiles). In the mutants, but not wild types, firing rate varied as a function of coupling strength for when all cells were included in the analysis; this relationship was also true for those cells that were significantly phase-locked to the mPFC slow gamma oscillation (Fig. 6b, c). These findings demonstrate a relationship between increased cross-frequency coupling and increased activity in mPFC neurons.

Stronger cross-frequency coupling also appeared to influence millisecond-level synchrony between mPFC neurons. Cross-correlations between simultaneously recorded neurons within the mPFC (Fig. 6d) were quantified by taking the height of the peak between –0.5 and 0.5 ms lag. The mean strength of cross-correlation was overall weaker in neuron pairs recorded from *Zdhhc8* ^*+/−*^ mice compared those from wild-type mice (Fig. 6e, f). In the mutants but not wild types, however, cross-correlation strength increased with increasing strength of vHPC theta–mPFC slow gamma coupling (Fig. 6e), such that at the highest levels of theta–gamma coupling, cross-correlations of *Zdhhc8* ^*+/−*^ neuron pairs were equal to those from wild types (Fig. 6f). These data suggest that the increases in long-range cross-frequency coupling seen in *Zdhhc8* ^*+/−*^ mice serve to increase activity and synchrony in mPFC neurons.

## Discussion

Here we report behavior-dependent increases in theta–slow gamma coupling between the vHPC and mPFC in *Zdhhc8* ^*+/−*^ mice, a mouse model of impaired spatial working memory. This enhancement is inversely correlated with choice accuracy of the task, and apparent only in choice phases of correct trials, suggesting that theta–slow gamma coupling reflects a compensatory mechanism to maintain working memory performance. Indeed, optogenetic and behavioral manipulations revealed that theta–slow gamma coupling is enhanced with increased task difficulty, regardless of the cause of this increase. Finally, enhanced vHPC theta–mPFC slow gamma coupling drives synchronous firing in mPFC neurons, suggesting that theta–slow gamma coupling can improve behavioral performance by modulating activity and synchrony within the mPFC. These findings provide evidence that long-range cross-frequency coupling plays a key functional role in hippocampal–prefrontal circuit function and working memory performance.

Our previous work demonstrated decreases in theta-frequency coherence between the vHPC and mPFC during performance of the T-maze task in *Zdhhc8* ^*+/−*^ mice^32^. A simplistic interpretation of this finding would suggest that decreased theta-frequency should lead to decreased ability of vHPC theta to modulate mPFC activity, including gamma oscillations, and therefore decreased theta–gamma coupling. Yet overall, vHPC theta–mPFC gamma coupling was increased in the mutant animals. Finding lower theta-synchrony yet increased theta–gamma coupling, suggests separable neurobiological substrates for the two forms of long-range synchrony. This is not the first time we have seen differential dependence of synchrony in multiple frequency ranges, even within the hippocampal-prefrontal circuit. Optogenetic inhibition of the direct hippocampal–prefrontal inputs disrupts gamma-frequency, but not theta-frequency synchrony between the vHPC and mPFC^27^. In *Zdhhc8* ^+/−^ mice, we reported impairments in vHPC axonal branching as well as reduced vHPC–mPFC theta synchrony, suggesting that theta-frequency synchrony between the vHPC and mPFC is disrupted by impairments in axonal development in these mice^32^. Some alternative, relatively intact mechanism seems to permit increased coupling of mPFC gamma to the vHPC theta, perhaps through more efficient engagement of inhibitory circuits known to be involved in the generation of gamma oscillations^34^.

The overall increase in theta–gamma coupling was associated with poor performance, both on a session-by-session and an animal-by-animal basis, implying that cross-frequency coupling might impair performance. Yet coupling was strongest during correct trials, arguing against such an implication. One way to reconcile these two findings would be if the enhancement in vHPC theta–mPFC gamma coupling represented an acute, adaptive response to impaired performance. To test this hypothesis, we examined the effect of two additional manipulations that impair performance in the T-maze task: the introduction of a longer delay between the sample and choice phase, and optogenetic inhibition of the direct vHPC-to-mPFC input during the sample phase. The latter manipulation has been shown impair behavioral performance as well as encoding of sample goal location by mPFC neurons^27^. Both manipulations increased theta–gamma coupling during the choice phase, consistent with the notion that increased vHPC theta–mPFC gamma coupling is a compensatory response aimed at preserving optimal performance.

There is a growing literature connecting theta–gamma coupling with successful behavioral performance, both in model organisms and in humans. Much of the animal literature is focused on theta–gamma coupling within the hippocampus, which increases during acquisition of item-context associations^19^ and correlates with performance in spatial memory^18^ and spatial alternation tasks^35^. In human subjects, intra-hippocampal theta–gamma coupling strength correlates with performance on a visual recognition working memory task^17^. Long-range cross-frequency coupling has been studied more in humans using electroencephalography; such studies have also shown associations between theta–gamma coupling strength and behavioral performance^36, 37^.

Our data demonstrated that theta–gamma coupling was associated with synchronous firing activity of mPFC neurons (Fig. 6), which may reflect temporally precise communication between neurons. Theta-nested gamma oscillations are thought to provide reference signal for coding schemes that require temporal coordination of firing activity^34^. Indeed, optogenetic theta frequency stimulation in the entorhinal cortex, which drove nested gamma oscillations, temporally organized action potential firing in concordance with nested gamma oscillations^13^. These data raise the possibility that theta–gamma coupling can facilitate synchronous firing activity which underlies successful working memory performance.

The current study builds on these findings by demonstrating enhanced, long-range theta–gamma coupling between the vHPC and mPFC associated with correct performance in the face of behavioral impairments. The data indicate that the hippocampal–prefrontal circuit is capable of dynamically optimizing its performance by upregulating the degree to which prefrontal gamma is time-locked to hippocampal theta, thus elevating firing rates and spike synchrony within the mPFC. While the precise mechanisms of this compensatory upregulation remain to be determined, the notion that impaired circuits can be fine-tuned through enhanced cross-frequency coupling is a compelling idea with important implications for both understanding differential disease susceptibility in individuals carrying identical genetic lesions, as well as for designing therapeutic neuropsychiatric interventions.

## Methods

### Animals

*Zdhhc8* ^+/−^ mice (*n* = 9) and their wild-type littermates (*n* = 8) were generated as described previously^38^ and bred on a mixed C57BL/6 × 129SveEv background. The effects of increased delay (*n* = 7) and optogenetic inhibition of vHPC terminals (*n* = 8 Arch and 6 EYFP mice) were examined in C57BL/6 mice obtained from Jackson Labs (Bar Harbor, ME). All mice were 3–6 months old at the time of the beginning of the experiments. All procedures were conducted in accordance with NIH regulations and approved by Columbia University and New York State Psychiatric Institute Institutional Animal Care and Use Committees.

### Surgery and recording

Mice were anesthetized with isoflurane (Butler Schein, Chicago, IL) and placed in a stereotaxic frame. A bundle of 13 twisted-wire stereotrodes (12.5 µm) were implanted in the mPFC (1.6 mm anterior to bregma, 0.3 mm lateral to midline, 1.4 mm below brain surface) and single tungsten wire field electrodes (75 µm) were implanted in the dHPC (1.94 mm posterior, 1.5 mm lateral, 1.4 mm ventral) and the vHPC (3.16 mm posterior, 3.0 mm lateral, 4.0 mm ventral). Skull screws attached above the frontal cortex and cerebellum served as reference and ground, respectively. All wires were connected to a 36-channel interface board anchored to a moveable Microdrive as previously described^25^. mPFC stereotrodes were regularly advanced to ensure that different cells were recorded in each session. Recordings were amplified, band-pass filtered (1–1000 Hz LFPs, 600–6000 Hz spikes), and digitized using the Neuralynx Digital Lynx system (Neuralynx, Tucson, AZ). LFPs were collected at a rate of 2000 Hz, while spikes were detected by online thresholds and collected at 32,000 Hz. All neural data in this study were from postcriterion sessions (see below). After all behavioral experiments were completed, electrolytic lesions (50 mA, 10 s) were created to mark electrode locations, which were visualized using a Nissl stain. There were no significant differences in the location of the electrodes by genotype (Supplementary Fig. 4).

### Optogenetic silencing of hippocampal–prefrontal pathway

Detailed methods for silencing are described before^27^. Briefly, AAV2/5 carrying hSyn-eArch-eYFP and hSyn-eYFP were used for opsin and control, respectively. Virus was targeted to multiple targets within pyramidal cell layer of ventral CA1 region (2 mediolateral rows at AP 2.95 and 3.25, with sites at ML/DV: 2.65/4.5, 3.0/4.3, 3.35/3.9, 3.7/3.3-2.9. An additional row was made at AP 3.1, with ML/DV sites at 2.8/1.55 and 3.15/1.7). All coordinates reported in mm, all AP and ML w.r.t. bregma, DV w.r.t. brain surface. Fiber coupled stereotrode bundles were then implanted bilaterally in mPFC, while LFP wires were implanted bilaterally in dHPC and vHPC.

### Working memory behavior

Animals were trained on a spatial delayed non-match to sample T-maze task, as described previously^25^. The maze consisted of a 55-cm-long center arm and two 32-cm-long goal arms, each 10 cm wide with 15 cm high walls. Each trial of the task consisted of a sample and choice phase. In the sample phase, a mouse ran down the center arm of the maze and was directed into one of the goal arms by the presence of a wall blocking the other goal arm. The mouse returned to the start box where it remained for a delay of 10 s (for all data except long delay sessions) or 90 s (for long delay sessions). In the choice phase, the mouse was required to enter the arm opposite to that visited during the sample phase to receive a reward. The behavior protocol began with 2 days of habituation to the maze for 10 min, followed by 2 days of shaping when animals were required to alternate between goal arms of the maze to receive food rewards. Training took place using 10 short delay trials daily. Once animals reached criterion-level performance (defined as performance of at least 70% correct per day for three consecutive days), recordings were obtained during daily sessions composed of 20–25 trials each session. While the *Zdhhc8* ^+/−^ mice took longer to achieve criterion, post-criterion performance did not differ by genotype (Supplemental Fig. 2b). For optogenetic inhibition, animals were run in 40–60 trials/session, with and without illumination of the mPFC during the choice, sample, delay, or whole trial on alternating trials as previously described^27^. Except where noted, all data come from center arm runs during performance after achieving criterion. The data presented in Fig. 5 are from whole trial off vs sample on trials only, and are taken from the choice runs; thus the data represent activity in choice runs without mPFC illumination, following sample runs that either had illumination (Light on) or did not have illumination (Light off).

### Data analysis

Data were imported into Matlab (MathWorks, Natick, MA) for analysis. Custom written scripts and scripts provided by K. Harris (University College London), C. Torrence and G. Compo (University of Colorado) were used.

Phase-power coupling: To examine the hierarchical relationship between theta frequency-range oscillations and gamma-frequency range oscillations, theta–gamma coupling was computed in a way similar to previously published methods^16, 19^. To analyze LFP changes in multiple frequency domains, the Morlet wavelet transform (1–150 Hz, a length of two cycles) was calculated with the wavelet software package (http://paos.colorado.edu/research/wavelets/software.html). LFP power in each frequency over time was represented by the square of absolute values of the result of wavelet convolutions. To calculate the phase of ongoing theta oscillations, LFP signals were filtered in the theta range (4–12 Hz) with a zero-phase-delay filter (filter0, provided by K. Harris and G. Buzsaki, New York University). The phase of the filtered LFP was then computed using the Hilbert transform. To measure the strength of theta–gamma coupling, theta phases were binned into *π*/50 intervals (0–360°) and the mean power of the low gamma (30–70 Hz) and high gamma (80–120 Hz) in each phase bin was calculated. The resulting values were input as weights to calculate the mean resultant length (MRL), which takes a value between 0 (no coupling) and 1 (perfect coupling). To calculate the comodulogram between low-frequency phase and high-frequency power, bands of low-frequency activity were extracted with a bandpass width of 0.5 Hz (centers at 4–20 Hz) and phase-power coupling was measured for wavelet power between 30–150 Hz.

Gamma peak analysis: LFP signal in the mPFC was filtered at fast gamma frequency (80–120 Hz) and slow gamma frequency (30–70 Hz) ranges. Then, a time series indicating the times of the peaks of the filtered signal was constructed, with the requirement that the peak times be separated by at least 100 ms from each other (i.e., just the highest peaks within 100-ms windows were used to avoid selecting multiple peaks within a theta cycle). Peak-averaged LFPs were obtained by averaging 200-ms epochs of the LFP raw signal in the vHPC centered at the time points corresponding to the gamma peaks. Theta phase distribution of peak gamma oscillations is shown as a histogram of the normalized number of peaks in each bin of theta phase.

Phase-phase coupling: Phase–phase coupling was analyzed as previously described^14, 16^. Phase coupling between two oscillators occur in an *n*:*m* ratio when there are *m* cycles of the “driven” oscillator for every *n* “stimuli”, which means that if there is a consistent *n*:*m* relationship, the difference between n*theta phase and m*gamma phase should have a consistent value. *n*:*m* coupling patterns were analyzed between theta range frequency and gamma range-frequency oscillations. The mean resultant length of the distribution of the difference between n*theta phase and m*gamma phase was calculated for variable *n*:*m* ratios shown in Supplementary Fig. 7.

Single unit analysis: For single units, neural signals were band-pass-filtered between 600 and 6000 Hz and waveforms that passed a threshold on either of the two stereotrode channels were digitized at 32,000 Hz. Waveforms were then sorted into single-unit clusters using KlustaKwik (by K. Hris, https://github.com/klusta-team/klustakwik/) followed by manual clustering. For the analysis of synchronous firing, units were required to fulfill the criteria: *L*-ratio ≤ 0.1 and isolation distance ≥10. There was no correlation between isolation distance and firing rate or phase locking, either the entire sample or for the subsample with isolation distances <10.

Spike cross-correlograms: To measure correlation of firing activity, we calculated spike train cross-correlograms (CCG) as described previously^39^. Our CCG is defined as:

$${\mathrm{CCG}} = \frac{{C_{12}}}{{T\sqrt {f_1f_2} }}$$

where *f*_1_ and *f*_2_ are the mean firing rates (in spikes per second) of neurons 1 and 2, and *T* is the duration of the spike train segments used to compute *C*_12_. *C*_12_ is a raw count of simultaneous spike trains over time in which the simultaneous spike train is defined as a spike pair occurring within 1 ms of each other. Dividing *C*_12_ by *T* changes the units of our CCG from raw coincidence count to coincidences per second. Normalizing by geometric mean avoids firing rate dependencies.

### Statistical analysis

Data are represented as mean ± s.e.m. Student’s *t*-tests after ANOVA was used for parametric statistics, whereas paired *t*-tests was used for paired comparisons. The significance of phase-locking of mPFC neurons to mPFC gamma oscillations were analyzed by Rayleigh’s test of circular nonuniformity. Watson–Williams test was used for the equality of the mean direction of theta–gamma coupling. To test the significance of correlations with multiple data points per animal, multiple linear regression analysis was conducted.

### Data availability

All data supporting the findings of this study are available within the article and its Supplementary Information or from the corresponding authors upon reasonable request.

## Electronic supplementary material

Supplementary Information
