# Supplementary material for: Hippocampal-prefrontal theta-gamma coupling during performance of a spatial working memory task
Source: Nat Commun. 2017 Dec 19;8:2182. doi: 10.1038/s41467-017-02108-9 (PMC5736608; doi:10.1038/s41467-017-02108-9)
Supplement: Supplementary file 1 — Supplementary Information [file 41467_2017_2108_MOESM1_ESM.pdf]

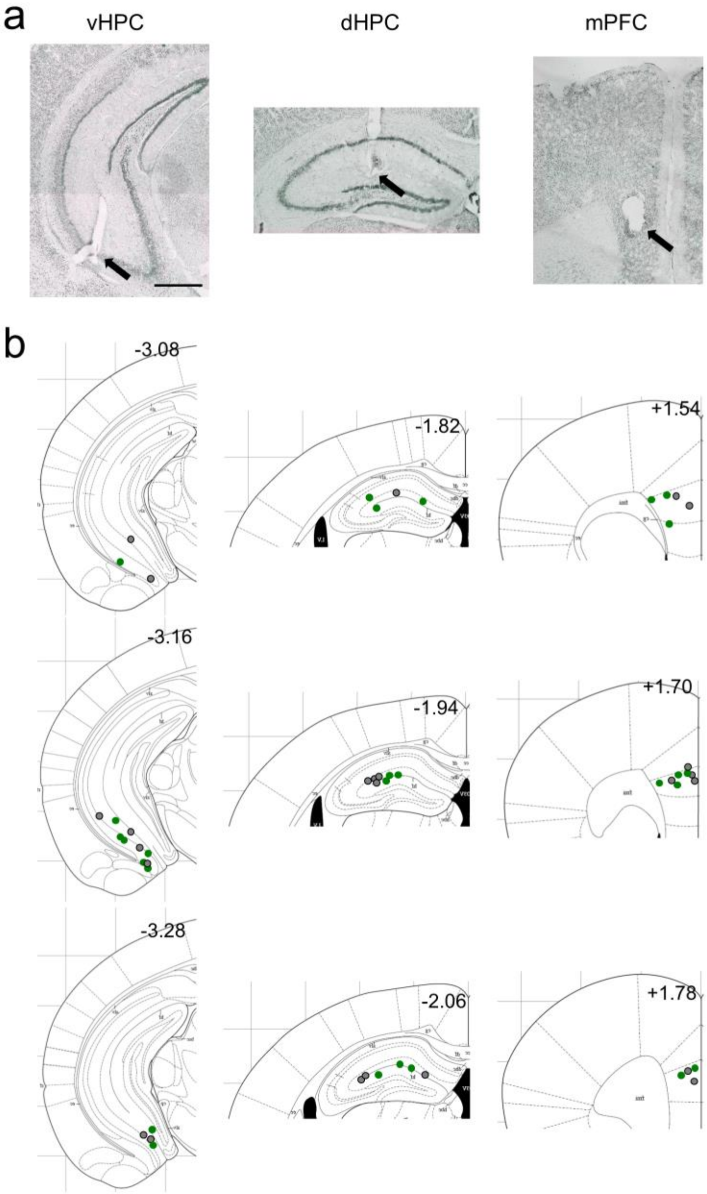

Supplementary Figure 1. Verification of electrode placements

(a) Representative images of Nissl-stained brain sections. Arrows indicate electrode tip locations. Bar shows 1mm.

(b) Electrode placements in wild-type (gray) and *Zdhhc8*$^{+/-}$ (green) mice.

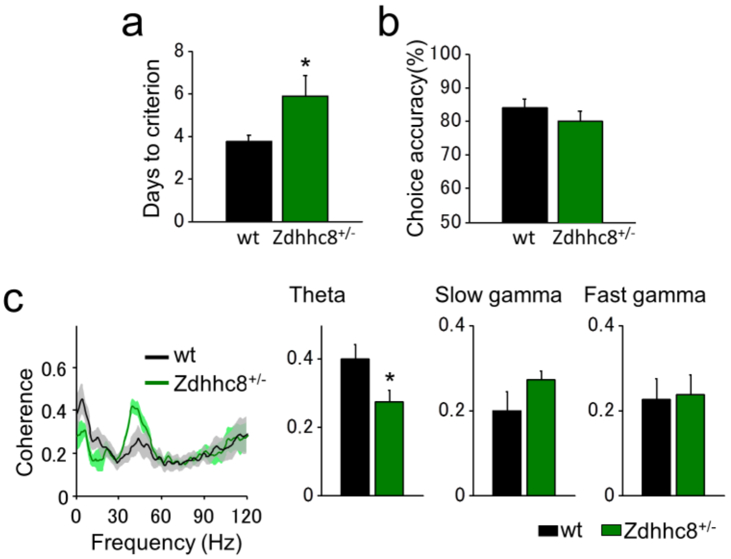

Supplementary Figure 2. *Zdhhc8* haploinsufficient mice showed impaired spatial working memory and hippocampal-prefrontal synchrony.

(a) Days to criterion in the T-maze spatial working memory task. *Zdhhc8*$^{+/–}$ mice took significantly longer time to acquire the task. *$p < 0.05$; Student's *t*-test.

(b) Choice accuracy after task acquisition in wild type and *Zdhhc8*$^{+/–}$ mice. There was no significant difference in choice accuracy after task acquisition by genotype, although *Zdhhc8*$^{+/–}$ mice show lower performance.

(c) Left: coherence spectra between the vHPC and mPFC. Right: averaged coherence in the theta-, slow gamma- and fast gamma-range frequency. Theta coherence was significantly disrupted in the mutant mice. *$p < 0.05$; Student's *t*-test.

Days to criterion (a) and theta-range coherence (parts of c) were shown in Mukai et al.[32] and are reproduced here for convenience.

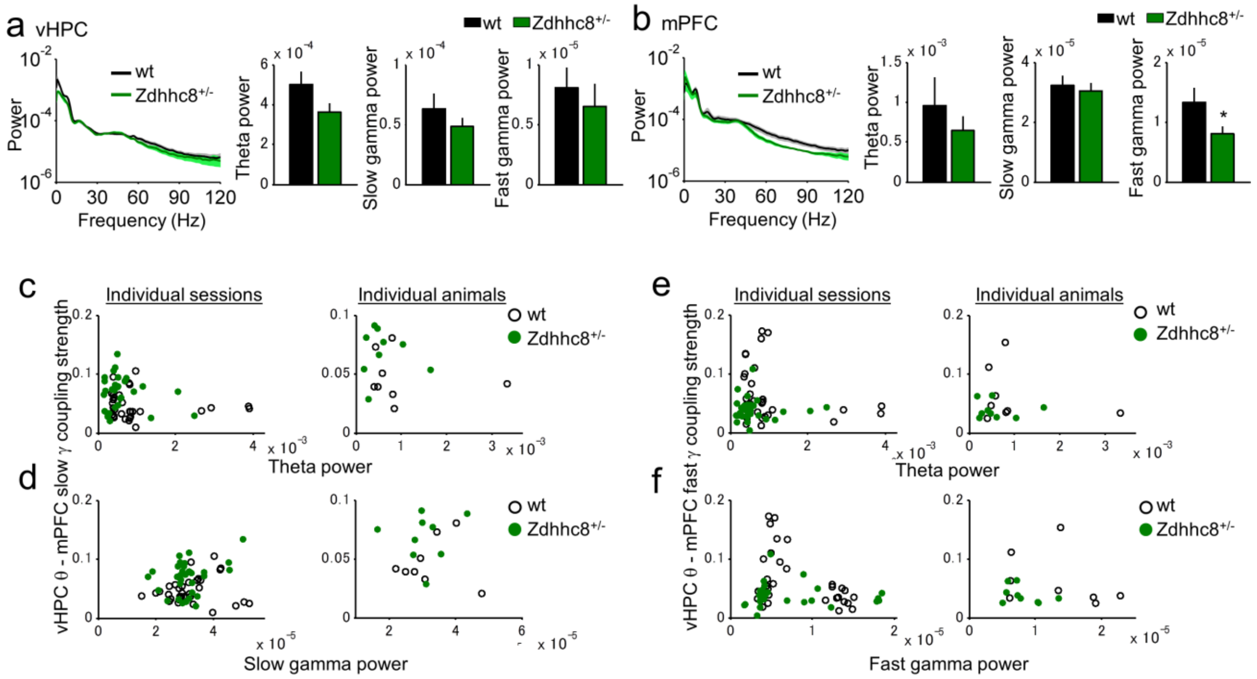

Supplementary Figure 3. Power spectra in the vHPC and mPFC during spatial working memory.

(a and b) Left: power spectra in the vHPC (a) and mPFC (b). Right: averaged power in the theta, slow gamma and fast gamma frequency ranges. There were no significant differences by genotype, except for fast gamma power in the mPFC (vHPC, theta; p = 0.13, slow gamma; p = 0.27, fast gamma; p = 0.51, mPFC, theta; p = 0.15, slow gamma; p = 0.64, fast gamma; p = 0.045; Student's t-tests).

(c - f) Scatter plots showing relationships between theta-gamma coupling and power. None of the correlations were statistically significant.

(c) vHPC theta-mPFC slow gamma coupling as a function of vHPC theta power (individual sessions: wt; R = -0.11, p = 0.54, *Zdhhc8*$^{+/-}$; R = -0.16, p = 0.39. individual animals: wt; R = -0.14, p = 0.74, *Zdhhc8*$^{+/-}$; R = -0.07, p = 0.86).

(d) vHPC theta-mPFC slow gamma coupling as a function of mPFC slow gamma power (individual sessions: wt; R = 0.087, p = 0.64, *Zdhhc8*$^{+/-}$; R = 0.25, p = 0.17. individual animals: wt; R = 0.062, p = 0.88, *Zdhhc8*$^{+/-}$; R = 0.11, p = 0.78).

(e) vHPC theta-mPFC fast gamma coupling as a function of vHPC theta power (individual sessions: wt; R = -0.22, p = 0.22, *Zdhhc8*$^{+/-}$; R = -0.01, p = 0.96. individual animals: wt; R = -0.23, p = 0.58, *Zdhhc8*$^{+/-}$; R = -0.11, p = 0.77).

(f) vHPC theta-mPFC fast gamma coupling as a function of mPFC fast gamma power (individual sessions: wt; R = 0.01, p = 0.17, *Zdhhc8*$^{+/-}$; R = -0.026, p = 0.88. individual animals: wt; R = -0.47, p = 0.24, *Zdhhc8*$^{+/-}$; R = 0.043, p = 0.91).

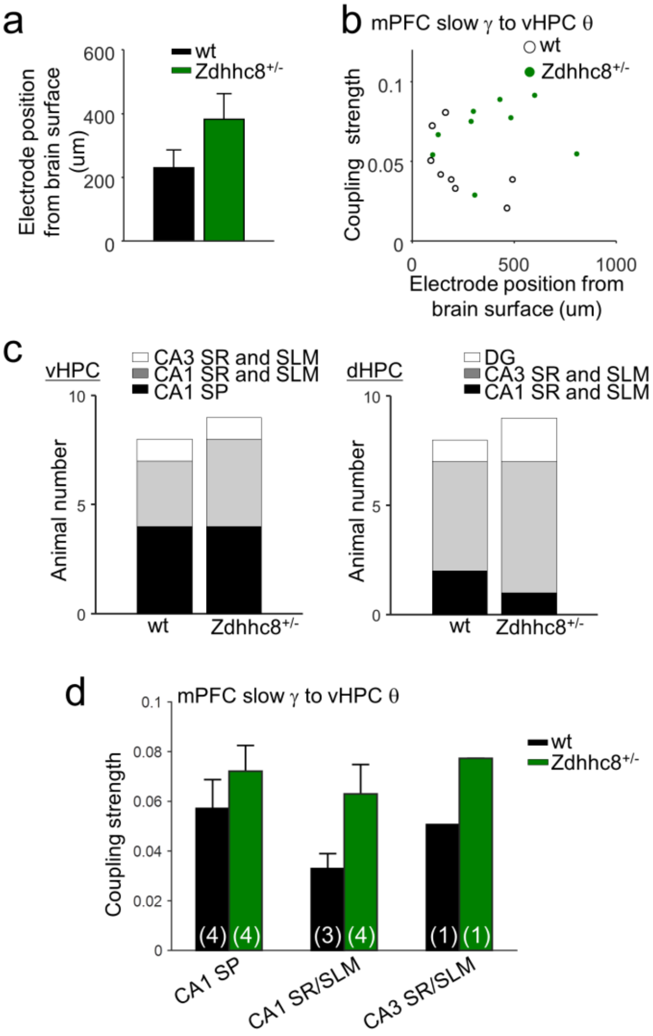

Supplementary Figure 4. Electrode positions did not affect strength of theta-gamma coupling.

(a) Averaged electrode positions in the mPFC from brain surface. Electrode positions did not differ by genotype ($p = 0.13$; Student's t-test).

(b) Scatter plot showing relationship between electrode position and strength of mPFC slow gamma to vHPC theta coupling. No significant correlation was seen in either genotype (wt; $p = 0.12$, *Zdhhc8*$^{+/-}$; $p = 0.62$).

(c) Distribution of electrode positions in the vHPC (left) and dHPC (right). DG: dentate gyrus, SP: stratum pyramidale, SR: stratum radiatum, SLM: stratum lacunosum-moleculare.

(d) Strength of mPFC slow gamma to vHPC theta coupling as a function of electrode position in the vHPC. Coupling strength seemed to be augmented in *Zdhhc8*$^{+/-}$ mice regardless of sub-region of the vHPC.

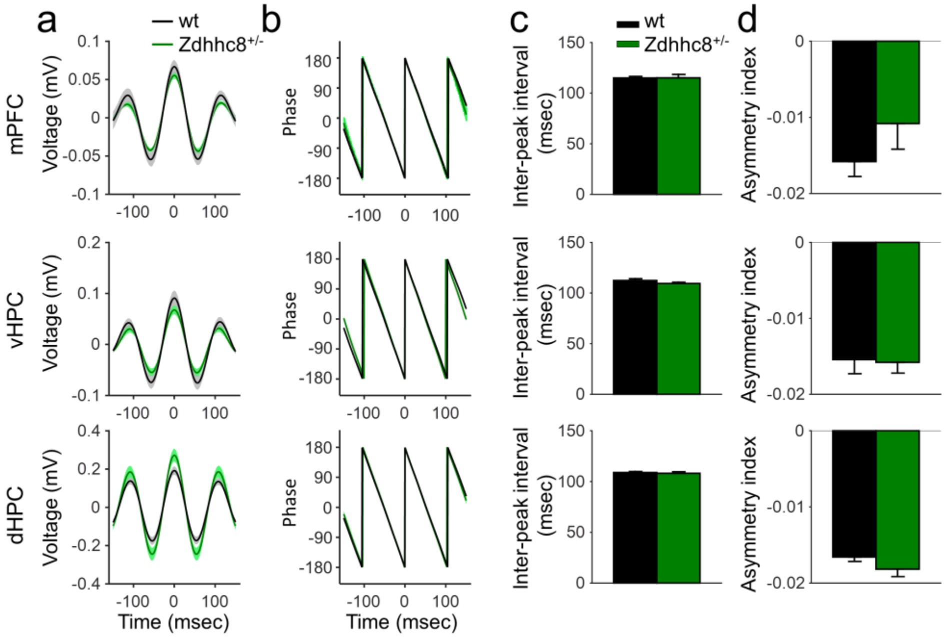

Supplementary Figure 5. Zdhhc8 haploinsufficiency did not affect theta phase shape in any regions.

(a and b) Averaged theta-range filtered traces (a) and phases (b) obtained by aligning the signals at the peak of theta oscillations. Top; mPFC, middle; vHPC, bottom; dHPC.

(c) Inter-peak interval of theta-range filtered local field potentials.

(d) Averaged asymmetry index. Asymmetry index is defined as the ratio of the duration of the ascending part and the duration of the descending part, expressed on a logarithmic scale, as previously described [14]. Negative values indicate shorter ascending and longer descending parts of theta.

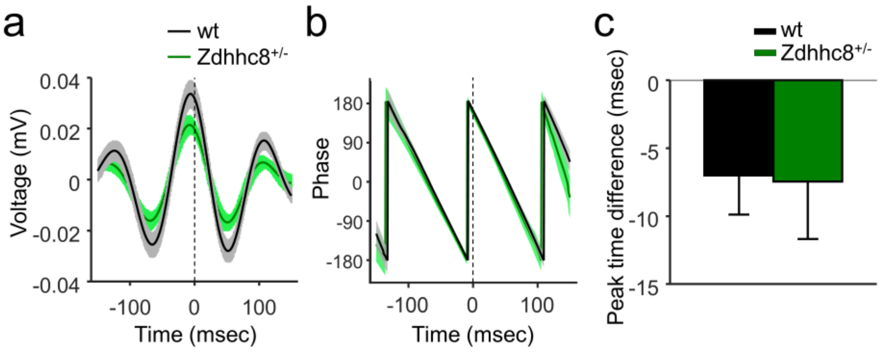

Supplementary Figure 6. Phase shifting of theta oscillations between the mPFC and vHPC.

(a and b) Averaged theta-range filtered traces (a) and phases (b) in the mPFC obtained by aligning the signals at the peak of theta oscillations in the vHPC.

(c) Averaged peak time difference of mPFC theta to vHPC theta. There was no significant difference in peak time difference by genotype.

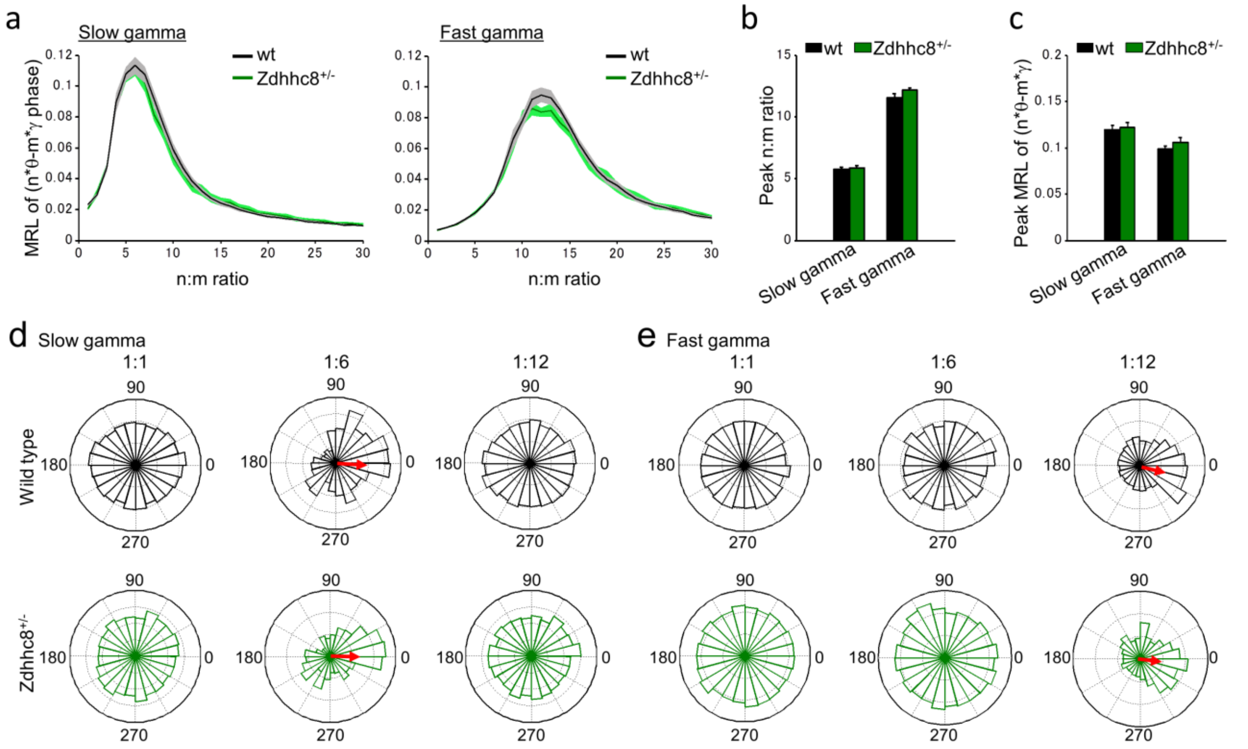

Supplementary Figure 7. Phase-phase coupling between vHPC theta and mPFC gamma is not affected by *Zdhhc8* haploinsufficiency.

(a) Mean resultant length (MRL) values from the distribution of phase differences, calculated for n*theta – m*gamma phase, as a function of the n:m ratio, for slow (left) and fast (right) gamma.

(b) n:m ratio that yields the peak MRL value averaged across animals, separately for wild type and *Zdhhc8*$^{+/–}$ mice. Peak n:m ratio was equivalent between genotypes both frequency ranges.

(c) Averaged peak MRL values for phase-phase coupling of slow and fast gamma. Peak MRL values do not differ by genotype.

(d and e) Examples of the distribution of the n:m ratio between theta and gamma phases for three different n:m ratios in slow gamma (d) and fast gamma (e) ranges. Only the distributions with 1:6 (slow gamma) and 1:12 (fast gamma) show unimodal distributions.

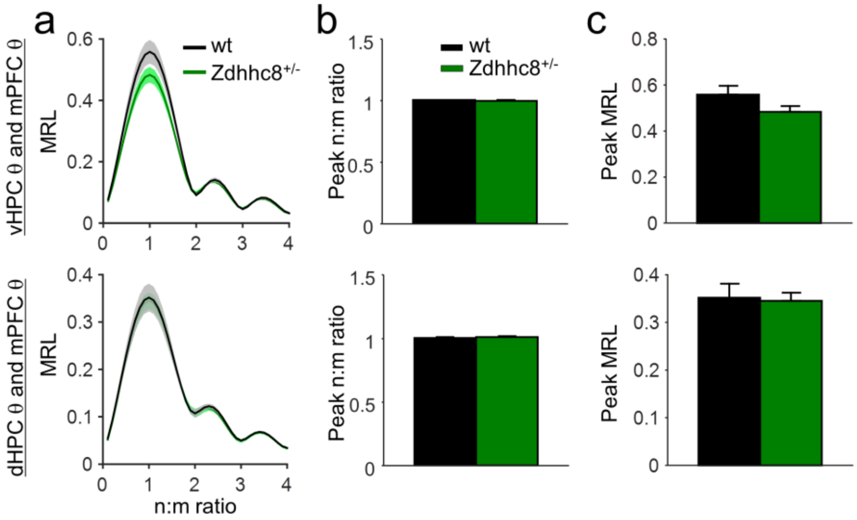

Supplementary Figure 8. Phase-phase coupling of theta oscillations between the HPC and mPFC.

(a) Mean resultant length (MRL) values from the distribution of phase differences, calculated for n*HPC theta – m*mPFC theta phase, as a function of the n:m ratio, for vHPC (top) and dHPC (bottom).

(b) Peak n:m ratio was equivalent between genotypes, regardless of region.

(c) Averaged peak MRL values for phase-phase coupling of theta oscillations. Peak MRL values did not differ by genotype regardless of region.

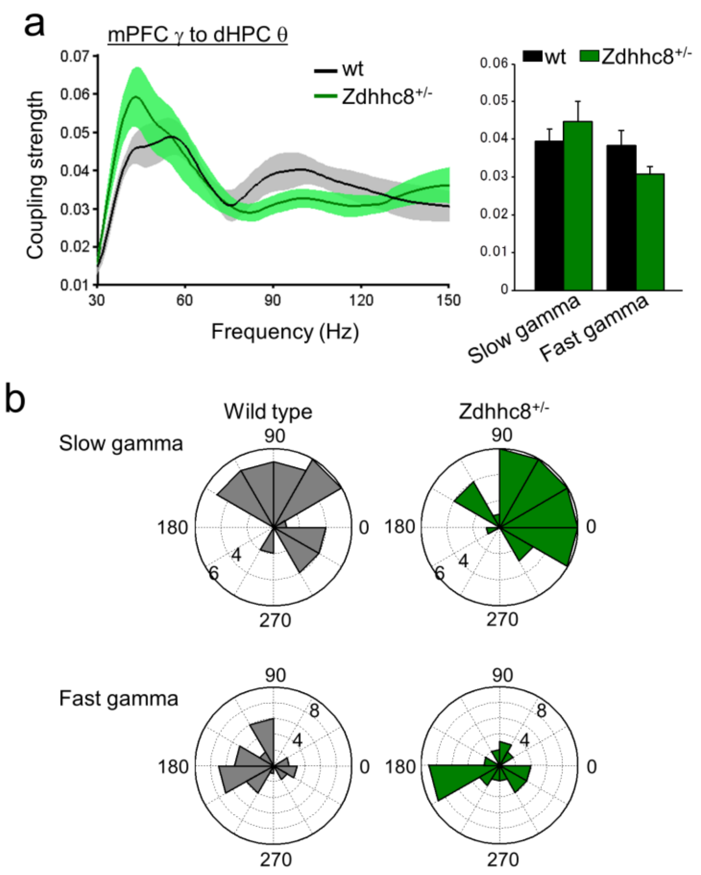

Supplementary Figure 9. Theta-gamma coupling between dHPC and mPFC.

(a) Left: strength of coupling between dHPC theta phase and mPFC gamma power as a function of power frequency in the mPFC. Right: averaged theta-gamma coupling in the slow and fast gamma ranges. Theta-gamma coupling did not differ by genotype (slow gamma; p = 0.42, fast gamma; p = 0.35, Student's *t*-tests). n=8, 9 for wt, *Zdhhc8*$^{+/-}$, respectively.

(b) Distribution of the mean direction of gamma power in the mPFC for the theta cycle phase in the dHPC. Mean direction did not differ by genotype (p > 0.05,Watson-Williams test).

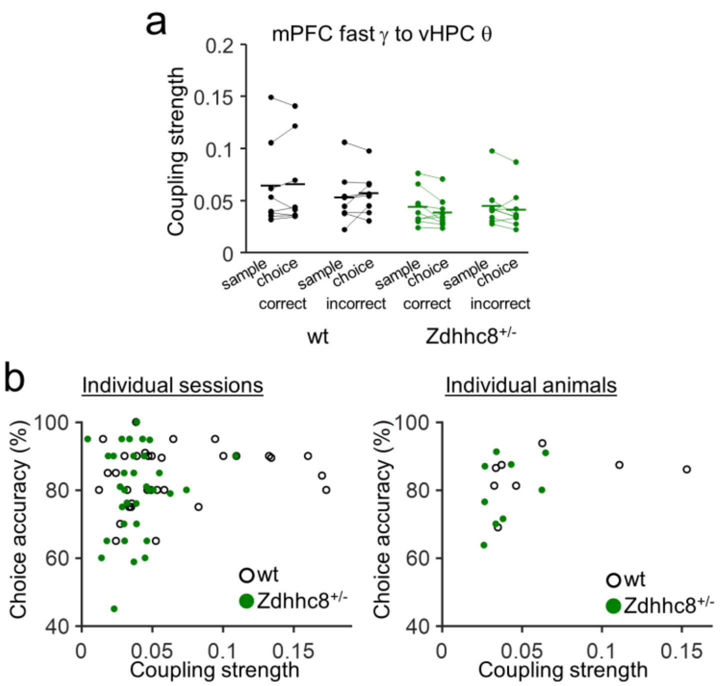

Supplementary Figure 10. Relationship of vHPC theta-mPFC fast gamma coupling with working memory performance.

(a) Strength of coupling in correct and incorrect trials. Coupling of mPFC fast gamma with vHPC theta did not differ by task phase in any groups (correct trials, wt; p = 0.72, *Zdhhc8*$^{+/-}$; p = 0.070. Incorrect trials, wt; p = 0.38, *Zdhhc8*$^{+/-}$; p = 0.20, paired t-test).

(b) Scatter plots showing the relationship between choice accuracy and coupling strength. Left: each session, right: each animal. There was no correlation between choice accuracy and coupling strength either in wild type and *Zdhhc8*$^{+/-}$ mice (Left: wt; R = 0.21, p = 0.24, *Zdhhc8*$^{+/-}$; R = 0.16, p = 0.24. Right: wt; R = 0.32, p = 0.43, *Zdhhc8*$^{+/-}$; R = 0.41, p = 0.27.)

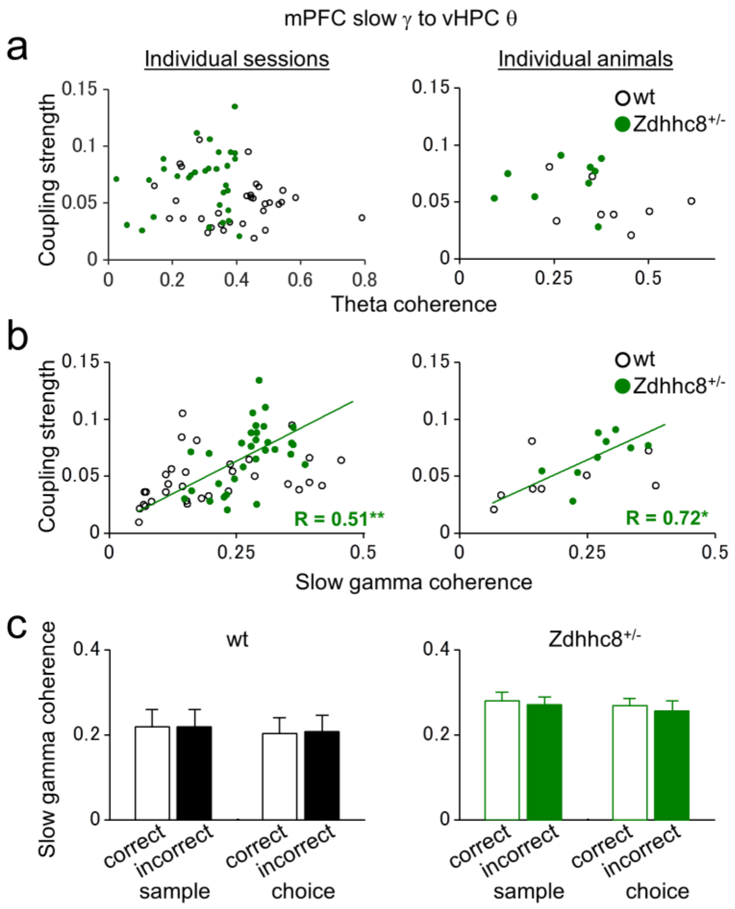

Supplementary Figure 11. Slow gamma coherence correlates with vHPC theta-mPFC slow gamma coupling, but does not differ between correct and incorrect trials.

(a and b) Scatter plots showing relationships of vHPC theta-mPFC slow gamma coupling with theta coherence (a) and slow gamma coherence (b). Coupling strength was positively correlated with slow gamma coherence between mPFC and vHPC. Left: wt; R = 0.37, p = 0.064, *Zdhhc8*$^{+/-}$; R = 0.51, p = 0.0020. Right: wt; R = 0.34, p = 0.41, *Zdhhc8*$^{+/-}$; R = 0.72, p = 0.028.

(c) Slow gamma coherence in sample and choice phases of correct and incorrect trials. There was no significant correct vs incorrect difference in any phase or between any groups (wt sample; p = 0.99, wt choice; p = 0.54, *Zdhhc8*$^{+/-}$ sample; p = 0.52, *Zdhhc8*$^{+/-}$ choice; p = 0.23). Paired *t*-tests.

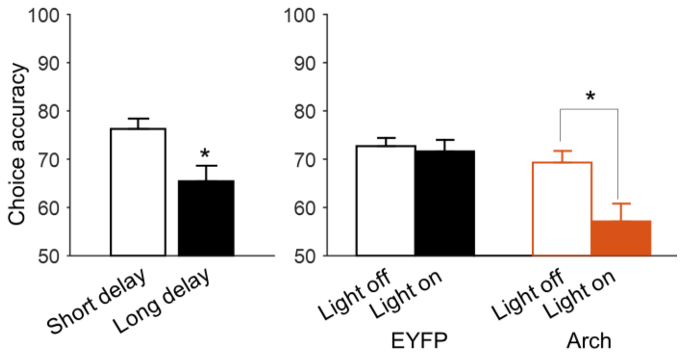

Supplementary Figure 12. Choice accuracy in the setting of increased difficulty in wild-type mice.

Choice accuracy was significantly decreased in longer delay trials (left) and optogenetic inhibition of vHPC-to-mPFC terminals (right). *$p < 0.05$; Student's t-test. Choice accuracy data from optogenetic inhibition was shown in Spellman et al.[27] and is reproduced here for convenience.

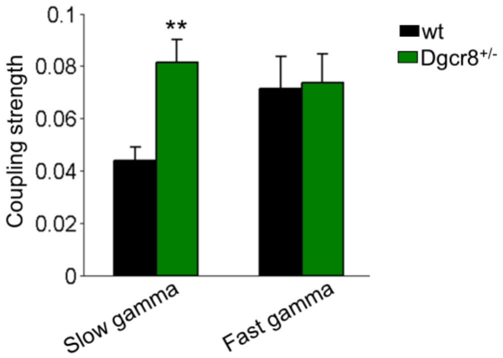

Supplementary Figure 13. vHPC theta-mPFC slow gamma coupling is augmented in correct trials of *Dgcr8*$^{+/-}$ mice.

Averaged theta gamma coupling in theta-slow gamma coupling and theta-fast gamma coupling, demonstrating significantly higher theta-slow gamma coupling in *Dgcr8*$^{+/-}$ mice (slow gamma; p = 0.0034, fast gamma; p = 0.95). **p < 0.01. Student's *t*-test. n = 8 wild type and n = 9 *Dgcr8*$^{+/-}$ mice.

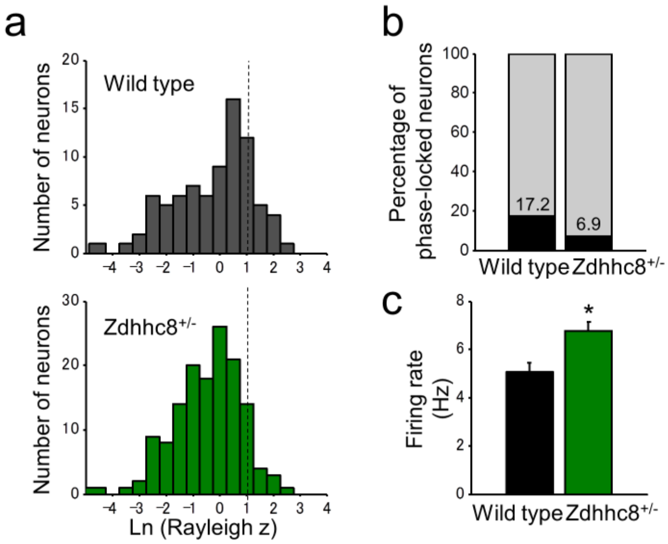

Supplementary Figure 14. Phase-locking of mPFC single units to local slow gamma.

(a) Distribution of Rayleigh z scores for computing strength of phase-locking of mPFC neurons to mPFC slow gamma oscillations. The distribution significantly differed by genotype ($p = 0.02$; Kolmogorov-Smirnov test). Dashed lines indicate the significance threshold ($p < 0.05$).

(b) Percentage of mPFC neurons significantly phase-locked to mPFC slow gamma oscillations. 17.2% for wild type mice. 6.9% for *Zdhhc8*$^{+/-}$ mice.

(c) Firing rates of mPFC neurons from wild type and *Zdhhc8*$^{+/-}$ mice. *$p < 0.05$; Student's *t*-test.
